# Pioneer transcription factor FoxA is positioned on hypersensitive nucleosomes at active enhancers

**DOI:** 10.1186/1756-8935-6-S1-P30

**Published:** 2013-03-18

**Authors:** Makiko Iwafuchi-Doi, Greg Donahue, Jason Watts, Isabel Cuesta, Kenneth S Zaret

**Affiliations:** 1 Epigenetic Program, Cell and Developmental Biology, Perelman School of Medicine, University of Pennsylvania, Philadelphia, PA, 19104-5157, USA

## 

The Nucleosome organization at gene regulatory sequences, such as at enhancers and promoters, is essential for understanding how genes are regulated. We have addressed how local nucleosome positioning and sensitivity are regulated in a tissue-specific manner focusing on pioneer transcription factor FoxA. FoxA can open a local domain of compacted chromatin *in vitro*, in the absence of ATP-dependent remodeling enzymes. Although micrococcal nuclease (MNase)-based genome-wide nucleosome maps have been developed extensively, many studies are subject to an overdigestion bias that may fail to map MNase-hypersensitive nucleosomes. We mapped the hypersensitive nucleosomes in mouse liver on genomic scale by carefully titrating the MNase digestion level. We found the hypersensitive nucleosomes were specifically located at active enhancers and promoters, and correlated with DNase l-hypersensitive sites. Furthermore, majority of FoxA2 binding events overlapped with the hypersensitive nucleosomes at active enhancers. We identified an amphipathic helix structure in C-terminal domain of FoxA that was required for the chromatin opening and the activation of target genes. We suggest that the pioneer transcription factor FoxA can organize nucleosome structures that are essential for gene activation.

